# A Modern Miracle

**Published:** 1870-09

**Authors:** 


### *A Modern Miracle.*

The *N. W. Christian Advocate* is responsible for the statement, (Aug. 10, 1870), that a few days since at the Oakington Camp Meeting, a Mr. Perkins of Cecil county, Md., who

"Had been laboring under a serious embarrassment of speech by stammering, which interfered with his usefulness, and he had entered into solemn covenant with God, that if He would restore to him his speech, he would solemnly engage to use his voice only to the glory of God, and to speak for the honor of his Master, whenever opportunity occurred. While he prayed there seemed to come upon him a peculiar unction from on high and he fell prostrate on the ground, and when enabled to rise again he began to praise the Lord with a free tongue, and came into one of the experience meetings, testifying that God had healed him soul and body. On every occasion thereafter during the meeting he spoke to the glory of God and with much liberty."

The physiological suggestions from this case are obvious.

